# The prognostic significance of CLEC‐2‐related gene signal in lung adenocarcinoma: A multicenter development and validation cohort

**DOI:** 10.1002/ctm2.100

**Published:** 2020-06-14

**Authors:** Chengmao Zhou, Lei Lei, Mu‐Huo Ji, Jian‐Jun Yang, Hongping Xia

**Affiliations:** ^1^ School of Medicine Southeast University Nanjing China; ^2^ Department of Anesthesiology and Perioperative Medicine First Affiliated Hospital of Zhengzhou University Zhengzhou Henan China; ^3^ School of Basic Medical Sciences & Sir Run Run Hospital & Key Laboratory of Antibody Technique of National Health Commission Nanjing Medical University Nanjing China

Dear Editor,

Non‐small cell lung cancer (NSCLC) is one of the most common carcinomas, in terms of both incidence and mortality. The standard treatment for patients with early‐stage NSCLC is radical resection, as tumor metastasis or relapse can lead to treatment failure and postoperative mortality.^1^ CLEC‐2 can promote cancer metastasis and cancer‐related thrombosis.^2^ Anti‐CLEC‐2 drugs are expected to prevent tumor metastasis and alleviate tumor‐related symptoms, thus extending survival in cancer patients.^3^ A model that integrates multiple CLEC‐2‐related cancer genes can provide better prognosis prediction accuracy than one that includes only one gene. Therefore, we used a Cox regression analysis to identify genes related to the prognosis from nine CLEC‐2‐related genes. We constructed a risk mode. Patients were divided into high‐ or low‐risk group by median risk score.

**Figure 1:**
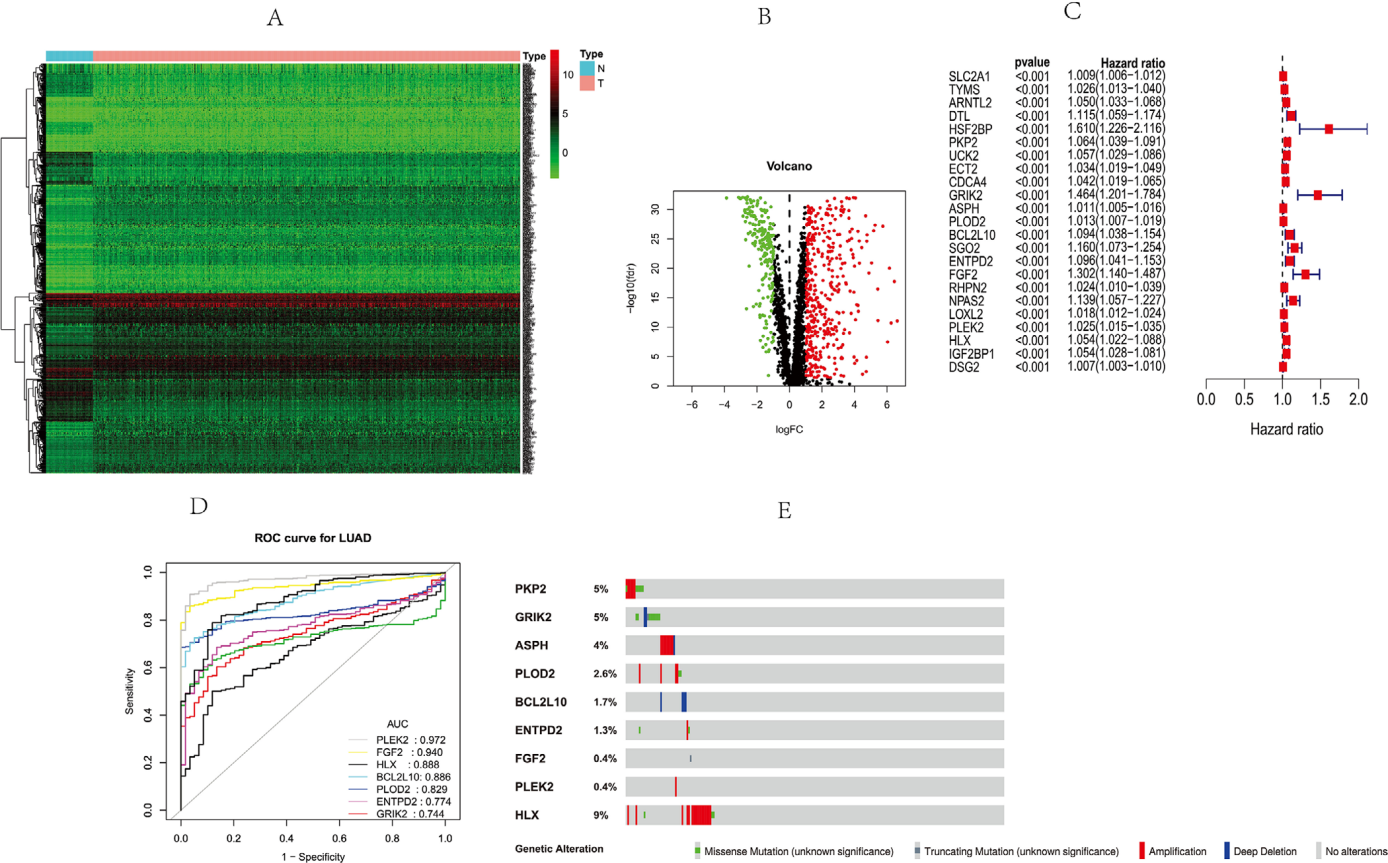
Differentially expressed CLEC‐2‐related genes and identification of hub CLEC‐2‐related genes. A, Heat map of differentially expressed CLEC‐2‐related genes in the TCGA. B, Volcano plot of differentially expressed CLEC‐2‐related genes in the TCGA. C, Single‐factor analysis for differentially expressed CLEC‐2‐related genes. D, ROC results of CLEC‐2‐related genes in diagnosis of lung adenocarcinoma. E, Mutation of CLEC‐2‐related genes in cBioportal database

We searched for CLEC‐2‐related genes in the GENE ONTOLOGY RESOURCE database (http://geneontology.org/). Six hundred and eighteen differential gene expression profiles in normal and tumor tissues in the TCGA‐LUAD cohort are shown in the heat maps and volcanoes (Figures 1A and 1B). Twenty‐four genes had statistically significant correlations with the TCGA‐LUAD's overall survival (OS) (Figure 1C). We calculated their regression coefficients with multiple COX regression analysis. Since the UCK2 gene was not available in the GSE68465 data set, only the remaining nine genes were included in the model. Among the nine genes, seven genes have area under the curve (AUC) values greater than 0.75 for diagnosing LUAD, of which PKP2 and FGF2 have the best efficacy, greater than 0.9 (Figure 1D).

Among the 230 patients who had taken the questionnaire, the number of patients with these nine gene mutations is 54.54 (23%) (Figure 1E). Risk score = PKP2*0.039302135+GRIK2*0.171236654+ASPH *0.00661721+PLOD2*0.00684938+BCL2L10*0.077131795+ENTPD2*0.056883973+FGF2*0.1691995447+PLEK2*0.011420101+HLX*0.058816735

**Figure 2:**
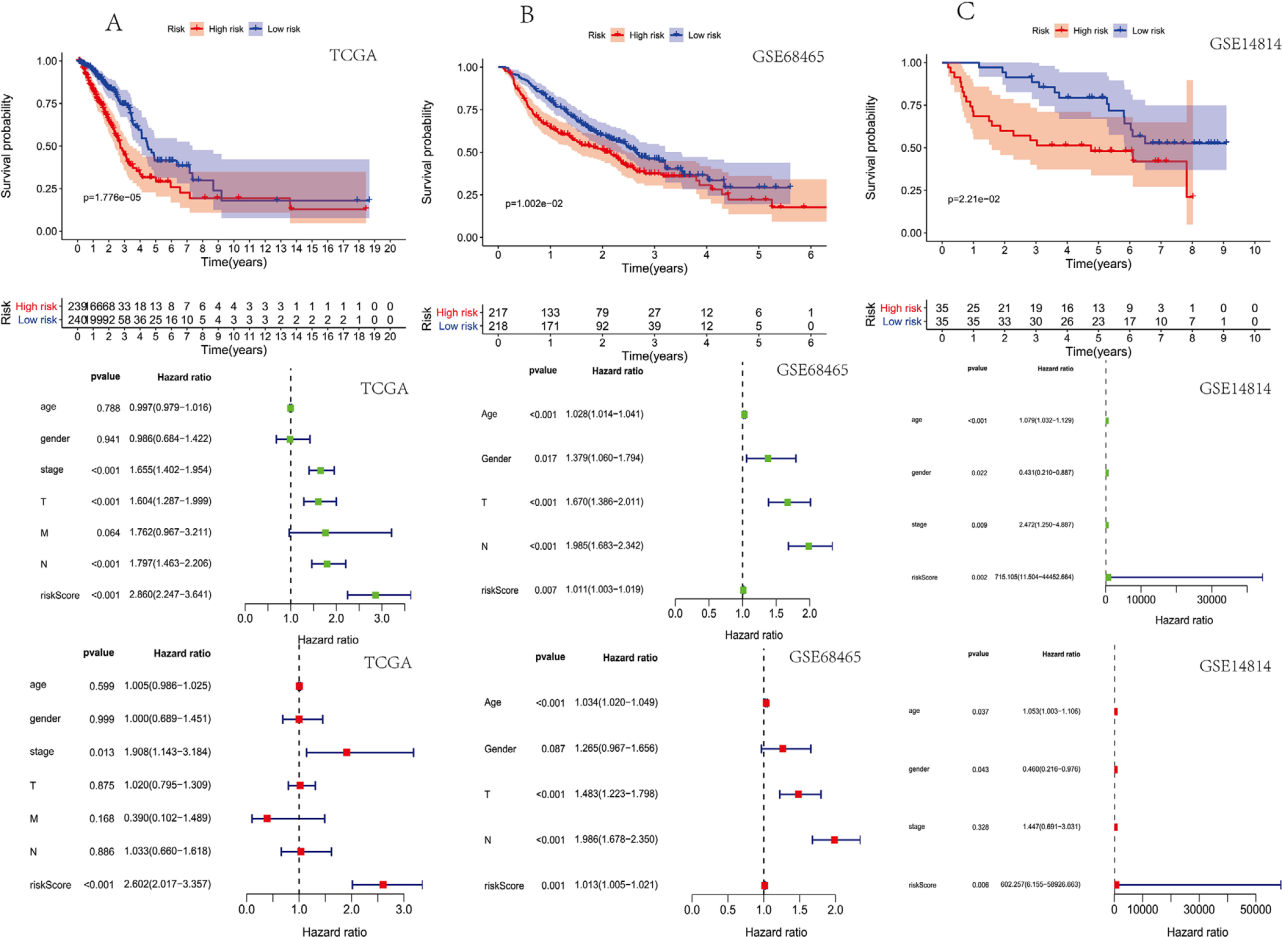
The prognostic value of nine CLEC‐2‐related risk scores. A, Kaplan‐Meier curves of overall survival based on the risk score and Single‐factor and multifactor analysis in the TCGA. B, Kaplan‐Meier curves of overall survival based on the risk score and Single‐factor and multifactor analysis in the GSE68465 cohort. C, Kaplan‐Meier curves of overall survival based on the risk score and Single‐factor and multifactor analysis in the GSE14814 cohort

In the TCGA‐LUAD cohort, Figure 2A shows the relationship between risk scores and survival times. Univariate analysis showed a statistically significant correlation between risk scores and OS (*P* = 1.776e‐05). The risk score was an independent prognostic factor (HR = 2.602, *P* < .001). The highest AUC for TCGA‐LUAD OS was a stage (AUC = 0.721) and risk score, (AUC = 0.718), respectively. AUC values for TCGA‐LUAD OS in these two queues still achieved good results (Figures 2B and 2C). We generated a nomogram to predict the likelihood of OS at the first, third, and fifth year by integrating nine CLEC‐2 gene signatures, age, sex, T, N, M, and TNM stages. The calibration curve showed that the actual and predicted survival rates matched, especially those of the 3‐year survival period. Moreover, we provided a free online web prediction tool that could provide personalized patient information, thus accurately predicting the individual survival prognosis (https://zcmnewnomogram.shinyapps.io/zcm-riskscore/). We analyzed whether our nine CLEC‐2 gene markers were related to immune infiltration in LUAD, such as B cells and neutrophils. The CLEC‐2‐related gene features are negatively correlated with the b cells (*r* = –0.154, *P* = 7.554e‐04); the CLEC‐2‐related gene features are positively correlated with the neutrophils (*r* = 0.124, *P* = .007). The CLEC‐2‐related gene features are positively correlated with the B7‐H3 (*r* = 0.235, *P* = 2.16e‐07) and PD‐L1 (*r* = 0.252, *P* = 2.36e‐08); the CLEC‐2‐related gene features are positively correlated with the neutrophils (*r* = 0.124, *P* = .007). The CLEC‐2‐related gene features are negatively correlated with the VEGFB (*r* = –0.151, *P* = 9.239e‐04) and positively correlated with the MKI67 (*r* = 0.343, *P* = 1.334e‐14). There were more TP53 gene mutations in the high‐risk group. We also conducted a subgroup analysis in an independent queue (GSE 72094). It showed that high risk was meaningless in the TP53 mutation subgroup. However, the risk score can effectively distinguish the OS rate in their wild group. There were differential copy numbers in nine CLEC‐2 gene markers with high and low risk, which get copy number in chromosomes 2, 3, 4, 7, 10, 11, 12, 15, 16, 17, 19, 20, and 22, and mitigated in 4, 5, 6, 10, 11, 12, 19, and 20. In the Human Protein Atls (HPA) proteomics database, the proteins of the genes constituting the risk score were also different. GSEA showed the potential mechanism of the high‐risk group may be mainly related to the P53 signaling pathway and cell cycle. Besides, among the other four cancers (brain lower grade glioma, bladder urothelial carcinoma, skin cutaneous melanoma, and thymoma), the prognosis of the two groups is also different according to the risk score (*P* < .05).

In recent years, reliable prognostic biomarkers have emerged, and these markers are changing the prognosis. With these biomarkers, high‐risk patient groups that may benefit from personalized treatment can be identified. However, the accuracy of the prognosis prediction remains unclear.^4^, ^5^ Now many studies have shown that CLEC‐2‐related genes may be associated with cancer.^6^, ^7^, ^8^, ^9^, ^10^

We constructed nine CLEC‐2 gene signatures based on the TCGA and GEO lung cancer cohorts (GSE 68465, GSE 14814, and GSE 72094). This genetic marker is an independent prognosis predictor. The mechanism may be related to the P53 signaling pathway and cell cycle. The nomogram, which is based on genetic and clinicopathological characteristics, can accurately predict lung cancer patients’ 1‐, 3‐, and 5‐year survival rates. At the same time, we also provided an online web prediction tool for nomograms (https://zcmnewnomogram.shinyapps.io/zcm-riskscore/). Our findings indicate that nine CLEC‐2 gene signatures may improve personalized treatment in clinical settings.
